# Exploring the metabolic signaling network of GFPT in cancer

**DOI:** 10.1038/s41420-025-02687-3

**Published:** 2025-08-19

**Authors:** Chibuzo Sampson, Pengfei Li, Yiqian Wang, Jing Liu, Jing Lv, Tian Xia, Hai-long Piao, Yegang Ma

**Affiliations:** 1 Department of Thoracic Surgery, Cancer Hospital of China Medical University, Liaoning Cancer Hospital and Institute, 110042 Shenyang, China; 2 Dalian Institute of Chemical Physics, Chinese Academy of Sciences, 116023 Dalian, China; 3 Department of Radiotherapy, The First Affiliated Hospital of Dalian Medical University, 116000 Dalian, China

**Keywords:** Cancer metabolism, Cancer microenvironment

## Abstract

Metabolic homeostasis is essential for cellular function in living organisms. In cancer cells, metabolic processes are reprogrammed to meet the energy demands and biosynthetic needs for rapid growth. This reprogramming enhances nutrient flux through the glycolytic pathway, supporting ATP production and branching into pathways that synthesize macromolecules required for cell proliferation. One critical branching pathway is the hexosamine biosynthesis pathway (HBP), which, driven by metabolic reprogramming, facilitates the synthesis of uridine-5’-diphospho-N-acetylglucosamine (UDP-GlcNAc), a glycosylation substrate. This pathway is regulated by the rate-limiting enzyme glutamine-fructose-6-phosphate transaminase (GFPT), a key controller of cellular UDP-GlcNAc levels and protein glycosylation. Dysregulation of GFPT is linked to metabolic disorders, like in diabetes, and it is also frequently upregulated in cancers. Given that GFPT plays a pivotal role in cancer metabolism, elucidating its regulatory interactions with other metabolic signaling pathways under metabolic stress is crucial to identifying therapeutic vulnerabilities in cancer. This review discusses the interaction network of GFPT with other metabolic pathways, its role in nutrient sensing, and the implications of GFPT deregulation in cancer.

## Facts

- The rate-limiting enzyme of hexosamkine biosynthetic pathway (HBP), GFPT, plays important roles in cellular signaling by controlling the level of HBP product UDP-GlcNAc to regulate protein glycosylation.
- The abnormal expression of GFPT has been detected in different cancer types, and GFPT participates in cancer progression through various manners.
- By promoting the flux of HBP, GFPT acts as a sensor of diverse cellular nutrients, and is involved in cancer metabolic reprogramming.
- According to its functions in cancer progression, targeting GFPT has been recognized as a promising strategy in cancer treatment.

## Introduction

Metabolic reprogramming is a hallmark of cancer, characterized by the metabolic alterations that malignant cells undergo to support rapid proliferation. First described by Otto Warburg, this phenomenon, known as “Warburg effect,” is marked by elevated glycolysis and glycolytic ATP production in normoxia, a process termed aerobic glycolysis [1]. Beyond glycolysis, cancer cells exhibit modifications in mitochondrial function that shift metabolism towards anabolic biosynthesis rather than ATP generation via the tricarboxylic acid (TCA) cycle. In contrast to quiescent cells, proliferating cancer cells must adapt to the challenging tumor microenvironment (TME), which is often marked by fluctuating oxygen levels, making the reliance on oxidative phosphorylation (OXPHOS) for ATP production less feasible [2]. Hence, cancer cells prioritize aerobic glycolysis to meet the energy demands and produce metabolic intermediates essential for the synthesis of macromolecules needed for cellular proliferation [3, 4]. To sustain this metabolic shift, cancer cells frequently acquire mutations that enhance nutrient uptake, particularly of glucose and glutamine [1, 5]. These nutrients supply carbon and nitrogen for biomass production; for example, glutamine provides a carbon source and amino groups crucial for nucleotide and protein synthesis, and it also fuels the hexosamine biosynthetic pathway (HBP). Furthermore, glutaminolysis contributes anaplerotic inputs to the TCA cycle and drives several biosynthetic pathways branching from the TCA [6]. Enhanced glucose uptake likewise promotes flux into anabolic pathways, including the HBP, pentose phosphate pathway, and serine biosynthetic pathway, all of which are vital for the anabolic synthesis of macromolecules in proliferating cancer cells [7].

**Fig. 1:**
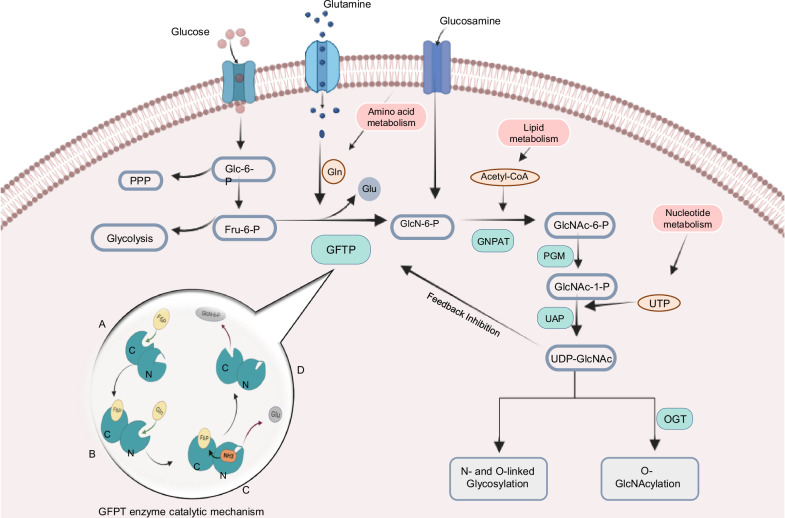
Hexosamine biosynthetic pathway (HBP). The HBP branches from glycolysis, utilizing ~2–5% of the glucose entering the cell. The enzyme GFPT, which has two catalytic domains (N-terminal and C-terminal), catalyzes the committed step of the HBP by converting fructose-6-phosphate (Fru-6-P) and glutamine (Gln) into glucosamine-6-phosphate (GlcN-6-P) and glutamate (Glu) as by-products. GlcN-6-P is then acetylated by glucosamine-6-phosphate N-acetyltransferase (GNPAT) to form N-acetylglucosamine-6-phosphate (GlcNAc-6-P), which is subsequently converted into GlcNAc-1-P by phosphoglucomutase (PGM3). Using UTP from nucleotide metabolism, uridine diphosphate N-acetylhexosamine pyrophosphorylase (UAP) catalyzes the conversion of GlcNAc-1-P to uridine diphosphate N-acetylglucosamine (UDP-GlcNAc), a substrate for O-GlcNAcylation (a post-translational modification) mediated by O-GlcNAc transferase (OGT), as well as for N- and O-linked glycosylation.

The hexosamine biosynthetic pathway (HBP) branches from glycolysis, beginning with the glycolytic intermediate fructose-6-phosphate. This nutrient-sensitive pathway, activated during metabolic reprogramming, is essential for supporting anabolic biosynthesis [8]. Approximately 2–5% of cellular glucose enters the HBP, leading to the production of uridine-5’-diphospho-N-acetylglucosamine (UDP-GlcNAc), a key glycosylation substrate. The HBP utilizes glucose, glutamine, acetyl-CoA, and UTP to produce UDP-GlcNAc, which is the precursor for both N-glycosylation and O-glycosylation of cellular proteins (Fig. 1). Glycosylation plays a crucial role in protein folding, cell signaling, immune response, inflammation, and epithelial-to-mesenchymal transition (EMT) [9, 10]. N- and O-glycosylation primarily occur in the secretory pathways of the endoplasmic reticulum and Golgi apparatus, involving the addition of complex glycans to proteins. In contrast, O-GlcNAcylation is a post-translational modification affecting nuclear and cytoplasmic proteins. Analogous to phosphorylation, O-GlcNAcylation is a dynamic process that transfers a single O-linked β-N-acetylglucosamine (O-GlcNAc) moiety to the hydroxyl groups of serine and threonine residues on proteins. Since O-GlcNAcylation often occurs on the same sites as phosphorylation, it directly or indirectly influences protein phosphorylation [11, 12]. Studies indicate that many phosphorylated proteins are also O-GlcNAcylated, highlighting a regulatory crosstalk between these modifications that modulates cellular signaling [11]. Nevertheless, while phosphorylation is mediated by multiple kinases, O-GlcNAcylation is cycled by a single enzyme pair: O-GlcNAc transferase (OGT), which adds the GlcNAc group, and O-GlcNAcase (OGA), which removes it. O-GlcNAcylation regulates various intracellular processes, and in cancer, it has been shown to modulate metabolic enzymes, nutrient transporters, as well as several oncoproteins and tumor suppressors involved in metabolic reprogramming [13–16].

The rate-limiting step in HBP is catalyzed by glutamine transaminase (GFPT), which is also known as GFAT, GFA (fungi ortholog), and GlmS (bacteria ortholog). GFPT mediates the transfer of an amino group from glutamine to fructose-6-phosphate (Fru-6-P), forming glucosamine-6-phosphate (GlcN-6-P), which is subsequently processed by downstream enzymes to produce UDP-GlcNAc (Fig. 1) [17]. The catalytic reaction is initiated by the binding of fructose-6-phosphate, which enhances the binding affinity for the second substrate, glutamine, and opens the hydrophobic ammonia channel [18–21], through which the ammonia released through glutamine hydrolysis is transported to the catalytic acceptor site within the isomerase domain (Fig. 1)[22].

## Featuers of glutamine-fructose-6-phosphate transaminase (GFPT)

**Fig. 2:**
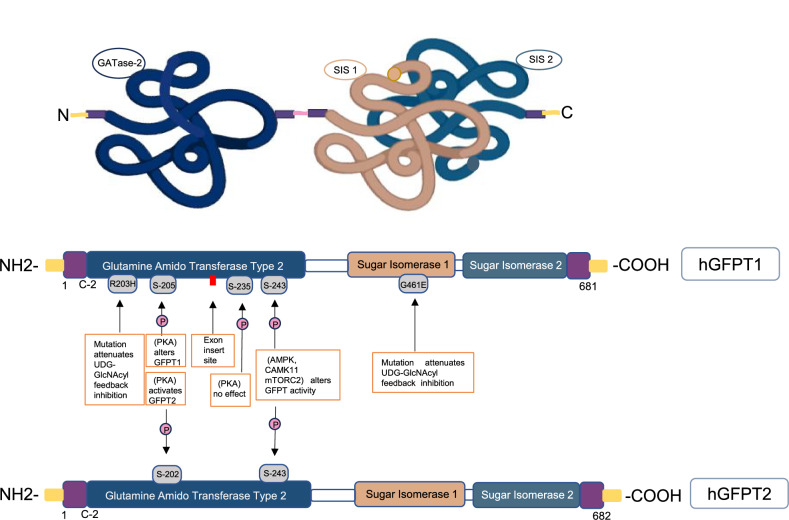
Structure domain and upstream activators of GFPT. GFPT contains two catalytic domains: the N-terminal glutamine amidotransferase type 2 (GATase-2) domain and the C-terminal sugar isomerase (SIS) domain, which functions as the synthase site. Several upstream regulators, including AMPK, PKA and mTORC2, modulate GFPT activity via phosphorylation.

GFPT is regulated by feedback inhibition from its end product, UDP-GlcNAc, whereas the bacterial ortholog is not susceptible to this inhibition [18, 23]. Additionally, the first catalytic product, GlcN-6-P, acts as a competitive inhibitor at the Fru-6-P binding site of GFPT [24]. In the hexosamine biosynthetic pathway, GFPT plays a pivotal role in controlling UDP-GlcNAc flux and cellular protein O-GlcNAcylation. Dysregulation of GFPT has been implicated in the metabolic disorders of many diseases, including cancer, diabetes, and neurodegeneration [25–27]. Structurally, GFPT contains two catalytic domains: the N-terminal glutamine amidotransferase type 2 (GATase-2) domain, which hydrolyzes glutamine to glutamate and ammonia, and the C-terminal sugar isomerase (SIS) domain, which facilitates the amination and isomerization of Fru-6-P to GlcN-6-P (Fig. 2) [28]. Mutations in *GFPT1* are associated with congenital myasthenic syndrome, a hereditary neuromuscular junction disorder [29, 30], while gain-of-function mutations in GFPT1 have been linked to extended lifespan in *C.elegans* [31]. Studies indicate that cancer cells upregulate GFPT1 to increase HBP flux, promoting abnormal protein O-GlcNAcylation [32]. Therefore, understanding the regulatory network associated with GFPT in metabolic reprogramming is crucial for identifying targetable metabolic vulnerabilities in therapeutic strategy development. In metazoans, there are two GFPT isoforms: GFPT1 and GFPT2, each with distinct tissue expression profiles [33]. GFPT1 is widely expressed, particularly in the pancreas, placenta, muscle, and testis, while GFPT2 is primarily found in the central nervous system [34]. A distinct GFPT1 isoform, featuring an 18-amino acid insertion at position 229, is specifically expressed in muscle tissue [35, 36]. GFPT1 and GFPT2 share ~79% sequence identity, and human GFPT1 and GFPT2 are consisted of 681 and 682 amino acids, respectively (Fig. 2) [37]. However, GFPT2 has lower sensitivity to UDP-GlcNAc feedback inhibition and does not strictly follow the bi-bi order of reaction, as it can hydrolyze glutamine even without Fru-6-P [38]. Throughout this review, we specify GFPT1 or GFPT2 when discussing individual isoforms and use “GFPT” to refer generically when the specific isoform is not indicated.

## GFPT-transcription factor regulatory network

**Fig. 3:**
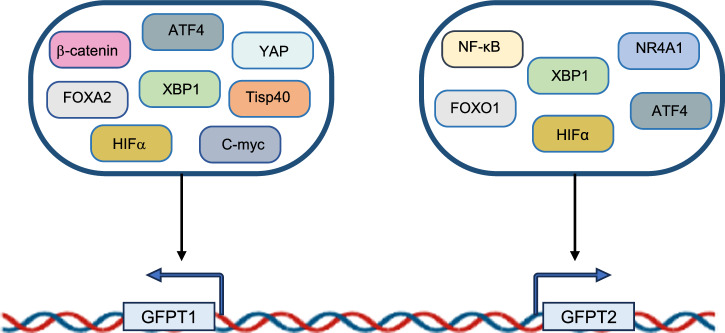
Transcriptional regulation of GFPT. Certain transcription factors drive the transcriptional upregulation of both GFPT1 and GFPT2, while others specifically target either GFPT1 or GFPT2 individually.

The enzymatic activity of GFPT is primarily regulated by protein kinases, while its mRNA and protein levels are controlled through transcriptional regulation (Fig. 3). As previously discussed, cellular stress triggers signaling pathways that induce GFPT expression by upregulating its transcription activators, to support metabolic adaptability under stress. Multiple studies have shown an intricate regulatory interplay between GFPT and transcription factors (Table 1).

**Table 1:** GFPT and metabolic signaling interaction.

Signaling pathway	GFPT isoform	Interacting protein	Outcome	References
PI3/mTOR	GFPT1	mTORC2	Maintain GFPT1 Phosphorylation. Promote GFPT1 expression.	[66, 67]
		C-myc	Transcriptional upregulation of GFTP1.	[55]
		PTEN	O-GlcNAcylation of PTEN leading to its Ubiquitination and degradation.	[97]
PI3/AKT/Mtor	GFPT1 and GFPT2	AKT	Activation of AKT and mTOR signaling Leading to cardiomyocyte hypertrophy.	[95, 96]
RAS/MAPK	GFPT1	MAPK/C-myc	Modulate the expression and protein level of GFPT1 to stimulate HBP flux.	[54]
		TAB1 and TLLS	Activation of p38 MAPK signaling to promote cell survival upon nutrient stress.	[114]
LKB1/AMPK	GFPT1	AMPK	Ser-243 Phosphorylation of GFPT1 and modulation of GFPT1 enzyme activity.	[65, 109]
cAMP/PKA	GFPT1	PKA	Ser 205 Phosphorylation of GFPT and modulation of GFPT1 enzyme activity.	[123]
	GFPT2	PKA	Ser 202 phosphorylation and enhanced GFPT2 activity.	[125]
UPR	GFPT1 and GFPT2	XBP1	Induces GFPT expression and cell survival in response to stress.	[46]
UPR	GFPT1 and GFPT2	ATF4	Induces GFPT expression and cell survival in response to stress.	[118]
NF-κB	GFPT2	NF-κB	Transcriptional expression of GFPT2 and feedback regulation of NF-κB.	[39]
WNT/β-catenin	GFPT1	β-catenin	GlcNAcylation and nuclear translocation and expression of β-catenin.	[40, 63]
HIF-1	GFPT1	HIF-1α	Increase HIF-1α expression and leading to HIF-1 signaling activation.	[41]
HIF-1	Not specified	HIF-1α	Augment the expression and activation of GFPT during hypoxic stress.	[44]
YBX-1	GFPT2	YBX-1	Promote YBX-1 O- GlcNAcylation, nuclear localization, and target gene expression.	
Hippo pathway	GFPT1	YAP	Promote YAP O- GlcNAcylation, activation, and nuclear localization.	[214]

The relationship of GFPT proteins with defferent pathways that have been reported are summarized here, including the interacting proteins, outcomes and references.

Metabolic remodeling enhances GFPT1-driven O-GlcNAcylation of key transcription factors, including NF-κB, HIFα, β-catenin, STAT3 and C-myc which play significant roles in cancer progression [39–43]. Conversely, transcription factors modulate the HBP and protein O-GlcNAcylation through GFPT expression. For example, hypoxic stress increases HBP flux by activating HIF-1α, which controls GFPT1 transcription [44, 45]. Similarly, endoplasmic reticulum (ER) stress initiates the unfolded protein response (UPR) via XBP1, a known transcriptional activator of GFPT [46]. Notably, while UPR signaling often upregulates GFPT2 expression in response to viral infection in human small airway epithelial cells [47], it specifically promotes GFPT1 expression in cardiomyocytes [46]. The reasons for this isoform-specific regulation remain unclear. However, it is proposed that GFPT2’s unique resistance to the UDP-GlcNAc feedback inhibition may contribute to the increased accumulation of UDP-GlcNAc post-infection. Moreover, the established association between GFPT2 and EMT could explain the preferential induction of GFPT2, as evidenced that GFPT2 is coexpressed with EMT-related proteins [40, 48]. In parallel, UPR-related transcription factor, Tisp40, has been shown to regulate GFPT1 expression and enhance protein O-GlcNAcylation thereby modulating cardiac dysfunction following ER stress or myocardial injury [49].

In cancer cells, chemotherapy resistance has been linked to the regulation of GFPT isoforms by specific transcription factors. For instance, the Forkhead box transcription factor FOXA2 promotes HBP-driven O-GlcNAcylation by transcriptionally activating GFPT1, consequently doxorubicin-induced apoptosis in hepatocellular carcinoma [50]. Several other oncogenic transcription factors, including NF-κB, HIF-1α, and c-Myc, also upregulate GFPT expression, which correlates with cancer progression. Notably, GFPT2 and NF-κB form a positive feedback loop by mutually enhancing each other to promote tumor development [39, 51]. In pancreatic ductal adenocarcinoma (PDAC) cells, the hypoxic microenvironment fosters metabolic adaptability and cell survival via HIF-1α-mediated GFPT1 expression [45]. Additionally, active GFPT2 expression is associated with c-Myc target genes, suggesting that HBP-induced O-GlcNAcylation stabilizes c-Myc in leiomyosarcoma [52]. Furthermore, c-Myc targets various enzymes in anabolic biosynthetic pathways, including GFPT, to support metabolic reprogramming and tumor growth [53, 54]. In glioblastoma, c-Myc-dependent activation of GFPT1 is linked to cancer cell proliferation, invasion, and metastasis [55]. Collectively, the regulatory interactions between transcription factors and GFPT1 are essential for reprogramming cellular metabolism to sustain growth under stress conditions.

## GFPT network in nutrient response

HBP integrates signals from various metabolic inputs—amino acid metabolism (glutamine), carbohydrate metabolism (glucose), lipid oxidation (acetyl-CoA), and nucleotide biosynthesis (UTP)—to produce UDP-GlcNAc, a critical substrate for protein glycosylation. As the rate-limiting enzyme of HBP, GFPT senses these nutrients to regulate pathway flux (Fig. 1). Nutrient fluctuations alter cellular metabolism and microenvironmental conditions, leading to adaptive changes in GFPT1 activity. For instance, elevated glucose levels are linked to increased GFPT activity (Fig. 1) [56, 57]. Hyperglycemia-induced pathologies, including diabetes and cardiovascular complications, have been associated with GFPT activation within the HBP pathway [58–60]. In cancer cells, glucose-fueled tumor progression and invasion have been linked to aberrant glycosylation resulting from heightened GFPT expression and increased HBP flux [61–63]. GFPT level, activity, and mRNA expression are regulated by nutrient-sensing kinases, growth factors, and transcription factors in response to nutrient availability. For example, under nutrient deprivation, AMPK and mTORC2 phosphorylate GFPT1 at serine 243 [64–66]. This regulation underscores the role GFPT as a nutrient sensor, which couples signals from metabolites to modulate HBP flux and cellular adaptation to metabolic stress.

Glutamine, an essential nutrient for cell growth, drives the HBP in rapidly proliferating cells. As a GFPT1 substrate, glutamine availability enhances GFPT1 activity, leading to increased GlcNAc levels and subsequent protein glycosylation (Fig. 1). The interaction between mTORC2 and GFPT1 is sensitive to glutamine levels [67]. Prolonged glutamine deprivation reduces mTORC2 activation and GFPT1 expression, while short-term glutamine withdrawal increases mTORC2 activity and sustains GFPT1 expression, highlighting the role of mTORC2 in modulating GFPT1 in response to glutamine homeostasis [67]. Glutamine thus sustains HBP flux during glucose limitation through an mTORC2-dependent mechanism [66]. In parallel, coordinated glucose and glutamine uptake fuels cell growth during metabolic reprogramming [68]. Notably, deprivation of glucose and glutamine, key substrates of the HBP pathway reduces UDP-GlcNAc levels and N-glycan branching, thereby skewing T cell differentiation toward pro-inflammatory TH17 cells at the expense of anti-inflammatory Treg cells [9].

GFPT1 is also responsive to lipid levels in addition to glucose and glutamine availability. Lipid metabolism generates acetyl-CoA, a crucial input into the HBP pathway (Fig. 1). Acetyl-CoA, a key component in fatty acid synthesis, drives lipid storage following excessive caloric intake. Overexpression of fatty acid synthase (FASN), a key enzyme in fatty acid synthesis, correlates with GFPT1 upregulation and enhanced colorectal cancer proliferation [69]. Thus, diets high in carbohydrates and fats stimulate GFPT1 expression and enhance HBP flux. Studies indicate that lipid consumption raises cellular O-glycosylation levels, likely linked to GFPT1-driven HBP flux [70–72]. Consistently, high-fat diet-induced obesity is associated with increased levels of O-GlcNAc-related proteins, including GFPT1 and OGT [73]. For instance, conjugated linoleic acid intake has been shown to elevate GFPT1 mRNA expression in porcine muscle, while palmitate and stearate boost protein O-glycosylation by upregulating GFPT1 [74, 75]. Moreover, saturated fat intake promotes GFPT2 expression in the mouse retina in an NR4A1-dependent manner [76].

Interestingly, nutrient stress, such as glucose or glutamine deprivation, can also enhance GFPT expression [77, 78]. Under such conditions, the unfolded protein response (UPR) is activated, upregulating GFPT as part of a stress adaptation mechanism [46, 77]. Reduced HBP-driven UDP-GlcNAc levels during nutrient stress typically impair glycosylation and glycan synthesis, leading to protein misfolding and UPR activation [79]. The resulting increase in GFPT1 enables cells to survive nutrient scarcity by enhancing their stress resilience. Additionally, HBP-induced O-GlcNAcylation regulates the expression of stress-responsive proteins, including specificity protein 1 (Sp1) and heat shock proteins [80, 81]. Overall, GFPT is directly and indirectly influenced by cellular nutrient levels and its nutrient response is critical for promoting cell growth.

## GFPT1 and cellular signaling network

**Fig. 4:**
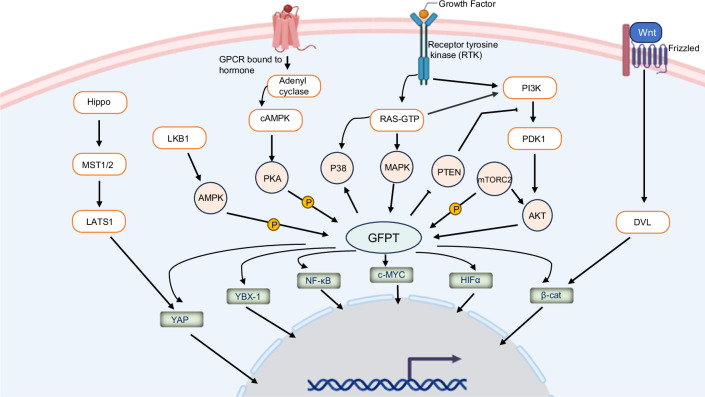
GFPT in metabolic signaling interaction network. GFPT interacts with multiple metabolic signaling pathways, including PI3K/mTOR, RAS/MAPK, Wnt/β-catenin, LKB1/AMPK, cAMP/PKA, and the Hippo pathway. Regulatory phosphorylation of GFPT by AMPK, PKA, and mTORC2 can either promote or inhibit its activity. GFPT-driven activation of transcription factors subsequently regulates the expression of their target genes.

**Fig. 5:**
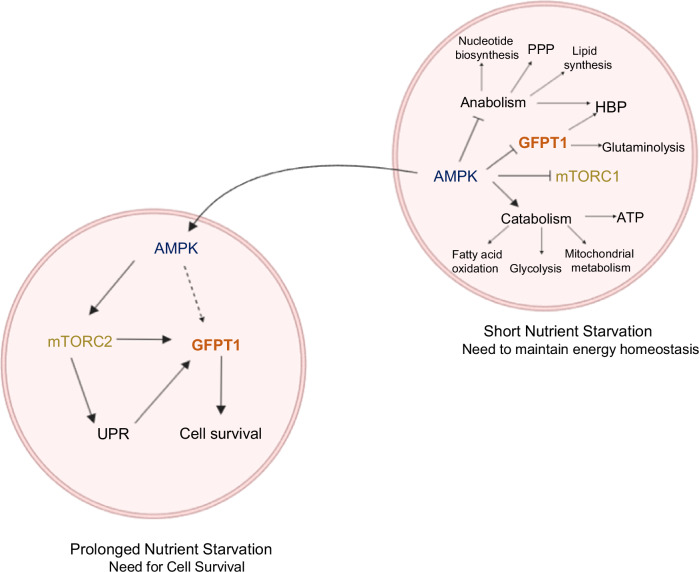
Regulatory interactions among AMPK, mTOR, and GFPT during acute and prolonged nutrient stress. Short nutrient starvation potentially inhibits GFPT1 directly via AMPK. In prolonged nutrient stress, GFPT1 activity is potentially maintained directly by mTORC2 and indirectly by AMPK.

Anabolic processes are fundamental to cancer progression, with the hexosamine biosynthetic pathway (HBP) playing a key role in driving protein glycosylation through its association with various oncogenic signaling networks. As the rate-limiting enzyme of the HBP, GFPT1 serves as a pivotal mediator, bridging oncogenic signals with metabolic pathways to support cellular anabolism. GFPT1 interacts with a range of molecular communicators, including kinases, immune checkpoint regulators, growth factors, and transcription factors, forming an extensive signaling network as outlined in Figs. 4 and 5 and summarized in Table 1.

## GFPT1 and the PI3K/AKT/mTOR pathway

The PI3K/AKT/mTOR pathway is a prominent oncogenic pathway that orchestrates cellular metabolic reprogramming and nutrient sensing [82, 83]. Activated by growth factors, this pathway facilitates metabolic remodeling in cancer by transmitting signals from PI3K (Phosphoinositide 3-kinase) through AKT (Protein Kinase B) to mTOR (mammalian target of rapamycin) and subsequently to downstream effectors and transcription factors, all of which drive cell growth [84]. Notably, GFPT1 has been implicated in interacting with PI3K/AKT/mTOR signaling, to impact the metabolic reprogramming associated with cancer as well as diabetic and cardiac diseases (Fig. 4) [85–87].

In glioblastoma, GFPT1 and mTORC2 form a regulatory network that adapts cancer metabolism to cellular nutrient levels [55]. Mechanistically, nutrient abundance activates mTORC2, which via c-Myc, enhances GFPT1 transcription and enzymatic activity. This upregulation increases HBP flux, promotes protein glycosylation, and reprograms cellular metabolism, collectively driving glioblastoma progression [55]. Acting as a glutamine sensor, mTORC2 responds to intracellular glutamine availability and promotes hexosamine biosynthetic pathway (HBP) flux by targeting GFPT1 through Ser243 phosphorylation [66]. Notably, this mTORC2-mediated GFPT1 phosphorylation at Ser243 persists at a prolonged but reduced amplitude during glucose starvation, which sustains GFPT1 expression and HBP activity even under nutrient limitation [66]. However, findings regarding this phosphorylation event are complex: Ser243 phosphorylation maintains the activity of GFPT1, which was not entirely abolished in SIN^-/-^ (a component of mTORC2) HeLa and MEF cells or in TORIN-treated cells under glucose starvation, suggesting that other kinases may also contribute to this regulation [66]. Additionally, basal phosphorylation at GFPT1 Ser243 continued even with limited AMPK activity, further implying alternative regulatory kinases [65].

Emerging evidence indicates that mTORC2 enhances GFPT1 expression through transcription factors like X-box binding protein 1 (XBP1) and c-Myc [55, 67]. Furthermore, AKT, a direct target of mTORC2 and upstream regulator of mTORC1, has been linked to GFPT1 expression and HBP activation via the AKT/XBP1 pathway, particularly in contributing to chemoresistance against agents like doxorubicin and camptothecin [88]. Intriguingly, GFPT1-driven O-GlcNAcylation also modulates AKT activation [89, 90], as lower O-GlcNAcylation levels, induced by pharmacological inhibition of GFPT1 or OGT, correlate with heightened AKT phosphorylation at Ser473 and Thr308 in acute myeloid leukemia (AML) cells [91]. This inverse correlation between O-GlcNAcylation and AKT activation has similarly been observed in pancreatic beta cells, kidney, adipocytes, and neurons [92–94]. In contrast, a direct correlation between GFPT-induced O-GlcNAcylation and AKT activation has been found in cardiomyocytes during hypertrophy, where increased O-GlcNAcylation driven by GFPT1 and GFPT2 promotes AKT-mediated hypertrophic growth [95, 96]. Additionally, GFPT1 acts as a negative regulator of the phosphatase and tensin homolog (PTEN), a known PI3K/mTOR inhibitor. Mechanistically, GFPT1 increases HBP flux and PTEN O-GlcNAc, which promotes PTEN ubiquitination and degradation, thereby stimulating PI3K/mTOR signaling and promoting the proliferation of cervical cancer cells [97]. This regulatory network positions GFPT1 as a key modulator in the interplay between nutrient sensing and oncogenic signaling, linking it to cancer growth and therapeutic resistance.

## GFPT-LKB1/AMPK pathway

AMPK (Adenosine Monophosphate-Activated Protein Kinase) is a central energy and nutrient sensor that regulates the cellular balance of ATP through the activation of catabolic pathways, including glycolysis, the TCA cycle, and fatty acid oxidation, in response to low glucose or ATP levels [98]. AMPK is a heterotrimeric kinase composed of three subunits: a catalytic α subunit, a scaffolding β subunit, and an AMP-sensing γ subunit [99]. Under nutrient scarcity, elevated AMP/ATP ratio leads AMP to bind the γ subunit, promoting phosphorylation of the α subunit at threonine 172 (Thr172) by upstream regulators such as LKB1 for AMPK activation [99, 100]. As a metabolic checkpoint, AMPK coordinates with other nutrient-sensing pathways to uphold metabolic homeostasis during nutrient stress [101].

The LKB1/AMPK-GFPT1 pathway was initially explored in adipocytes, where increased HBP flux via GFPT1 overexpression enhances AMPK phosphorylation and activation and consequently promots fatty acid oxidation (Fig. 4) [102]. This effect is mediated by O-GlcNAcylation of AMPK in adipocytes. However, reports on AMPK activation through O-GlcNAcylation are mixed: while O-GlcNAcylation has been linked to increased AMPK activity, acute inhibition of O-GlcNAc cycling—leading to higher O-GlcNAc levels—appears to suppress AMPK activation [103–106]. Interestingly, inhibiting GFPT1-mediated O-GlcNAcylation in bladder cancer cells enhances AMPK activation and induces autophagy [104]. These varied findings suggest additional regulatory factors or context-specific roles for O-GlcNAcylation in AMPK regulation.

The direct interaction between AMPK and GFPT1 shows that glucose scarcity activates the LKB1/AMPK pathway, leading to AMPK-mediated phosphorylation of GFPT1 at Ser243 (Fig. 2) [64, 65]. This phosphorylation reportedly inhibits GFPT1 activity and reduces HBP flux, impacting processes like VEGF-induced angiogenesis [65], ER-induced apoptosis [107], and anti-hypertrophic responses [108]. Conversely, another study suggests that Ser243 phosphorylation enhances GFPT1 activity [109], aligning with mTORC2-mediated GFPT1 regulation under nutrient stress. The discrepancy underscores a complex regulatory network that demands further exploration, discussed in detail in the following sections.

## GFPT1-KRAS/MAPK pathway

KRAS (Kirsten Rat Sarcoma virus), a well-known proto-oncogene, is frequently mutated and activated in several cancers [110]. KRAS signaling drives tumor proliferation, survival, and aggressiveness through downstream activation of MAPK (Mitogen-Activated Protein Kinase) and PI3K pathways [111, 112]. A major outcome of KRAS activation is metabolic reprogramming, which enhances nutrient uptake and anabolic biosynthetic pathways in cancer cells [113]. One key metabolic shift in KRAS-mutant cancers is the increased hexosamine biosynthetic pathway (HBP) flux, which boosts protein O-GlcNAcylation and N-glycan biosynthesis.

Oncogenic KRAS is pivotal in directing glycolytic intermediates into anabolic pathways, specifically the pentose phosphate and HBP pathways [54, 112]. KRAS signaling modulates GFPT1 expression, thereby increasing HBP flux and protein glycosylation in pancreatic ductal adenocarcinoma (PDA) cells [54]. The transcription factor Myc, a downstream effector of KRAS, is known to regulate GFPT1 and other metabolic genes in PDA cells [54]. Importantly, GFPT1 has been shown to modulate p38 MAPK activation independently of the HBP-mediated glycosylation pathway. Specifically, GFPT1 interacts with and glutamylates TAB1 (Transforming Growth Factor-β-Activated Kinase 1 Binding Protein 1), thereby promoting the recruitment and activation of p38 MAPK to support lung cancer cell survival under conditions of glucose deprivation (Fig. 4) [114]. Furthermore, KRAS-induced lung cancer aggressiveness is associated with GFPT2 expression [115]. The transcription factors Snai1 and Twist1, both working as key regulators of epithelial-mesenchymal transition (EMT), are implicated in upregulating HBP enzymes, especially GFPT2, in KRAS-mutant lung cancer [115]. Similarly, KRAS and LKB1 co-mutations in non-small cell lung cancer (NSCLC) promote GFPT2 expression, which correlates with poor prognosis [116]. The potential mechanism is that since the LKB1/AMPK axis generally inhibits HBP flux, loss of LKB1 indirectly promotes HBP activation via GFPT2, whose expression and activated is maintained by KRAS [65, 116].

KRAS also responds to glutamine availability by regulating genes involved in amino acid metabolism, including those mediating glutamine utilization such as GFPT1, especially in nutrient-limited NSCLC [117]. This regulation involves the transcription factor ATF4 (Activating Transcription Factor 4), which targets GFPT expression in response to endoplasmic reticulum stress [118]. The enrichment of unfolded protein response (UPR) genes regulated by KRAS under low-glutamine conditions may explain ATF4 activation during nutrient stress [117]. Given that KRAS-mutant tumors with elevated HBP activity rely on this pathway for survival, HBP enzymes, particularly GFPT, present a promising targetable vulnerability in KRAS-driven cancers [119].

## GFPT-cAMP/PKA pathway

Protein kinase A (PKA), or cAMP-dependent protein kinase, is activated directly by cAMP and subsequently promotes the release of glucose and free fatty acids through gluconeogenesis, glycogenolysis, and lipolysis to support cellular energy requirements and is associated with metabolic reprogramming and cancer cell survival under nutrient stress, suggesting its potential interplay with the glucose-sensing HBP pathway (Fig. 4) [13, 120–122].

Two consensus PKA phosphorylation sites, RRGS for Ser205 and KKGS for Ser235 have been identified in human GFPT1 (Fig. 2) [123, 124]. The RRGS motif, conserved across eukaryotes, corresponds to the Ser202 site in GFPT2, while the KKGS motif is not conserved and does not exist in GFPT2 [124–126]. Phosphorylation at Ser205 in GFPT1 or Ser202 in GFPT2 by PKA has been shown to modify GFPT activity, while no significant impact was observed for phosphorylation at Ser235 (Fig. 2) [123–125]. Specifically, phosphorylation at GFPT2 Ser202 is known to enhance GFPT2 activity, yet reports on GFPT1 Ser205 phosphorylation have been inconsistent, showing both inhibition and activation effects [123, 124]. A study addressing this discrepancy with a phospho-mimic S205D mutant and an R203H gain-of-function mutant in GFPT1, finding that phosphorylation at Ser205 disrupts UDP-GlcNAc feedback inhibition, thereby sustaining GFPT1 activity [127]. This regulation suggests that PKA-dependent Ser205 phosphorylation positions the glutaminase and isomerase domains of GFPT1 in a configuration that prevents UDP-GlcNAc-mediated inhibition. Thus, at high UDP-GlcNAc concentrations, Ser205 phosphorylation serves as an activating mechanism by disrupting feedback inhibition, while at low concentrations, it reduces GFPT1 catalytic activity, acting as an inhibitory regulation [127].

Since PKA typically promotes glucose availability by inducing glycogenolysis and gluconeogenesis [121], it is plausible that PKA could also negatively regulate GFPT1 to sustain cellular glucose homeostasis. However, in nutrient-deprived cancer cells, PKA activity is linked to protein N-glycosylation and glutamine metabolism, which are crucial for cancer cell survival, suggesting a positive association with HBP flux and potentially GFPT activation [122]. Additional studies are required to elucidate the role of PKA-mediated GFPT1 phosphorylation and to determine if this function exhibits tissue specificity.

## Dissecting the regulatory crosstalk of mTORC2 and AMPK on GFPT

The regulation of GFPT1 by mTORC2 and AMPK under nutrient starvation conditions is complex and involves nuanced control via Ser243 phosphorylation. While both mTORC2 and AMPK phosphorylate GFPT1 at this site, the functional outcome of this modification remains contentious. Eguchi et al. and Zibrova et al. observed that AMPK-mediated phosphorylation at Ser243 suppresses GFPT1 activity [64, 65]. In contrast, Li and colleagues reported that AMPK phosphorylation boosts the synthesis of GlcN-6-P by GFPT1, even though the glutaminase activity is reduced, suggesting that Ser243 phosphorylation may not necessarily result in a decrease in overall GFPT1 function [109]. Supporting this, recent findings indicate that mTORC2-mediated Ser243 phosphorylation enhances GFPT1 activity, and sustains hexosamine biosynthetic pathway (HBP) flux during nutrient scarcity [66]. Additionally, while Eguchi et al. noted an activity reduction with AMPK-mediated phosphorylation, this suppression persisted in a GFPT1 S243A mutant, suggesting that AMPK-dependent inhibition of GFPT1 activity may not be solely due to Ser243 phosphorylation [64].

Studies indicate that AMPK negatively regulates O-GlcNAcylation [108, 128], possibly due to its impact on UTP levels. For example, 2-deoxyglucose treatment, which activates AMPK, results in a pronounced reduction of UTP during nutrient stress, yet elevated GlcNAc-P levels suggest GFPT1 remains active under these conditions [66]. Additionally, since HBP flux could be affected by other downstream enzymes within the pathway, these factors should be considered when using O-GlcNAcylation levels to assess GFPT1 activity. GFPT is known as a critical stress-response protein that supports cell survival during nutrient deprivation, and its activity is expected to increase under nutrient stress.

During acute glucose deprivation, GFPT1 may be downregulated via AMPK to curb anabolic processes, while prolonged nutrient stress likely upregulates GFPT1 to support cell survival (Fig. 5) [67, 78]. Notably, AMPK facilitates mTORC2 activation to promote cell survival under glucose-starved conditions [129]. Although mTORC2 activation is initially measured during acute glucose withdrawal, its expression and activity are even higher under prolonged starvation and are linked with UPR activation, leading to increased GFPT1 expression [67, 129]. Thus, an AMPK-dependent signal may support sustained GFPT1 expression during nutrient deprivation. Although protein expression alone does not always equate to increased activity, elevated GFPT1 levels during nutrient stress have been correlated with tumor progression and poor patient prognosis, indicating that GFPT1 expression is crucial for its role in promoting cancer growth and survival [78]. Overall, GFPT1 is also regulated by additional kinases, metabolites, and feedback inhibition, making it essential to dissect the combined effects of these regulatory mechanisms on GFPT1 activity during short-term and prolonged nutrient stress, as well as their variability across different cell lines.

## GFPT and cancer

### Dysregulation of GFPT in cancer

**Fig. 6:**
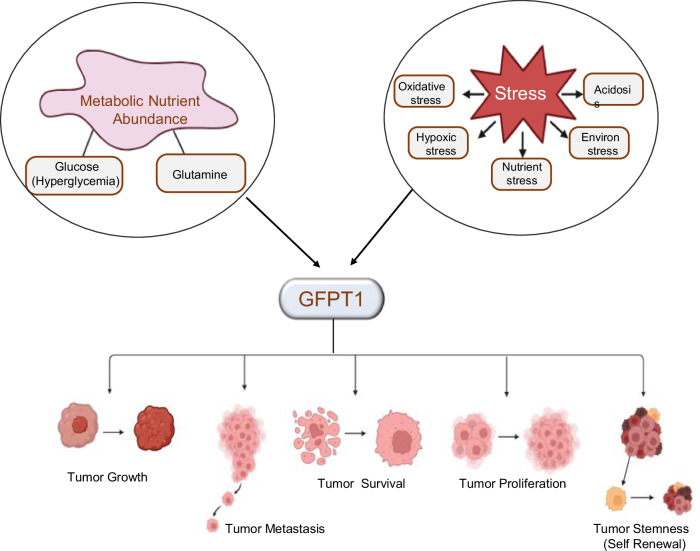
Dysregulation of GFPT in cancer. GFPT acts as a nutrient and stress-responsive protein, activated under conditions of nutrient abundance and cellular stress. In cancer, GFPT upregulation is closely linked to tumor progression and metastasis.

The tumor microenvironment is characterized by multiple stress factors, such as nutrient depletion and hypoxia, which drive metabolic rewiring to support cancer cell growth, survival, and progression. With the emerging focus on cancer metabolic reprogramming, the HBP has gained attention due to its critical role in stress response. Aberrant HBP flux in cancer, driven by dysregulation of GFPT, is associated with various aspects of cancer progression, including enhanced proliferation, survival, self-renewal, and metastasis (Fig. 6 and Table 2).

**Table 2:** Dysregulation of GFPT1 in Cancer.

GFPT isoform	Cancer type	Expression status	Role in cancer	References
GFPT1/GFPT2	Lung cancer	Upregulation	Tumor growth and survival. Associated with EMT, tumor invasion, migration and metastasis.	[51, 114]
GFPT1/GFPT2	Colon Cancer	Upregulation	Promote EMT tumor growth, invasion and metastasis. Associated with tumor microenvironment and immune infiltration.	[39, 61, 131]
GFPT1/GFP2	Pancreatic ductal Adenocarcinoma (PDA)	Upregulation	Promote tumor aggressiveness lymph node metastasis and stemness. Associated with poor overall survival.	[132, 134, 142, 171]
GFPT1	Glioblastoma	Upregulation	Accelerate growth of tumor and proliferation.	[55]
GFPT1	Prostate Cancer	Upregulation	Tumor proliferation.	[42]
GFPT1	Cervical Cancer	Upregulation	Enhances tumor proliferation.	[97]
GFPT1	Acute Myeloid Leukemia (AML)	Upregulation	Promote proliferation and inhibit cancer cell death.	[91]
GFPT1	Hepatocellular Carcinoma	Upregulation	Promote proliferation, Migration and invasion. Critical for TNM stage and poor overall survival.	[133]
GFPT1	Gastric Cancer	Downregulation	Associated with EMT, invasion and migration.	[162]
GFPT2	Gastric Cancer	Upregulation	Promote tumor invasion and pathological stage. Associated with immune infiltration and poor prognosis.	[148]
GFPT2	Serous Ovarian Cancer	Upregulation	Promote EMT, Cell invasion and migration.	[40]
GFPT2	leiomyosarcoma	Upregulation	Associated with enhanced glucose uptake, c-MYC stability and poor patient outcome.	[52]
GFPT2	Urinary Bladder Cancer (UBC)	Upregulation	Associated with immune infiltration tumor pathological grade and metastasis.	[163]
GFPT2	Breast Cancer	Upregulation	Promote tumor growth, EMT, and stemness. Associated with Claudin low breast cancer.	[140, 154]
Not specified	Cholangiocarcinoma	upregulation	Increases tumor invasion, migration, and EMT.	[62]

GFPT1 has been reported to participate in the progression of different cancer types. Here we list the expression alterations of GFPT1 in diverse cancer types as well as its function in cancer development.

### GFPT in cancer cell proliferation and stemness

The growing interest in the role of HBP and glycosylation in tumors has led to a focus on GFPT, though its precise role in cancer is still indistinct. Several evidences support the essential role of GFPT in cancer metabolic reprogramming [32]. Elevated glycolysis and the Warburg effect in proliferating cells increase the diversion of glycolytic intermediates into the HBP for enhanced glycosylation. This process relies on the upregulated expression and activation of GFPT, which catalyzes the committed step of the HBP pathway [130]. Consequently, abnormal glycosylation due to dysregulated GFPT gives cancer cells a proliferative edge.

The expression of GFPT is elevated in many malignancies [52, 131–133] and correlates with tumor growth and poor prognosis, making it a promising therapeutic target. Both in vivo and in vitro studies have demonstrated that GFPT is essential for cancer cell proliferation [54, 97], and pharmacological inhibition of GFPT has been shown to suppress tumor growth and cell viability [61, 119, 134]. Similarly, GFPT depletion in cancer cells reduces proliferation and progression [55, 135]. Specifically, GFPT1 has been identified as a key metabolic driver in PDAC cells [54] and as a prognostic marker linked to tumor size in triple-negative breast cancer [136]. Meanwhile, GFPT2 plays a critical role in tumor metabolic reprogramming in lung cancer [137], acts as a neoplastic marker in tenosynovial giant cell tumors (TGCT) [138], and functions as an androgen receptor-inducible target involved in prostate cancer metabolic reprogramming [42].

GFPT has been implicated in cellular protection and defense against oxidative stress-induced cell death [77, 139, 140]. It has been observed that UDP-GlcNAc addition mitigates UPR-induced cell death and aids the survival of KRAS-transformed cells during glucose deprivation [141]. This protective mechanism is propagated through GFPT-mediated HBP activity, leading to increased glycosylation, improved protein folding, and subsequent inhibition of UPR activation. Thus, targeting the GFPT1/HBP pathway appears a promising manner for promoting cell death in proliferating cancer cells experiencing nutrient stress.

Furthermore, GFPT1 plays a significant role in promoting self-renewal and stemness of cancer cells. High GFPT1 expression has been observed in cancer stem cells (CSCs) within pancreatic cancer [142] and hepatocellular carcinoma stem cell-like populations [143]. Interleukin-8-induced CSC development in colon and lung cancer cells relies on GFPT1-mediated O-GlcNAc modifications, demonstrating the critical involvement of the HBP pathway in cancer stemness [144]. Besides, GFPT-driven HBP activation is associated with cancer chemoresistance [88]. Upregulation of GFPT influences the efficacy of doxorubicin and cisplatin in inducing cancer cell apoptosis [50, 145]. A proteomic study identified GFPT1 as a predictive marker for tamoxifen resistance in estrogen receptor-positive breast cancer [146]. A recent study demonstrates that gemcitabine administration induces GFPT2 expression and thereby promotes the invasion of pancreatic cancer [147]. Meanwhile, GFPT2 expression has been linked to increased sensitivity to certain classes of chemotherapeutic agents [148], suggesting that GFPT expression could serve as a predictive marker for drug response. In summary, GFPT is a central player in cancer metabolic reprogramming, supporting cancer cell proliferation, survival, stemness, and drug resistance.

### GFPT in epithelial-mesenchymal transition in cancer cell

Epithelial-mesenchymal transition (EMT) is a hallmark of cancer metastasis that promotes invasive and aggressive phenotypes in tumor cells. During EMT, the epithelial marker E-cadherin (CDH1) is downregulated, leading to a loss of cell-cell adhesion, while mesenchymal markers, such as N-cadherin (CDH2), are upregulated to enhance cell migration [149]. This metabolic reprogramming, which supports the transformational phenotype in cancer, is closely tied to the GFPT-driven HBP flux [150].

Several studies have demonstrated a substantial link between the HBP pathway and EMT [32, 151]. The EMT-HBP connection is implicated in the metastasis of various cancers, including lung cancer [152], melanoma [153], ovarian cancer [40], colorectal cancer [39], and breast cancer [154]. Since the HBP pathway supplies the UDP-GlcNAc required for the synthesis of glycan, which plays a fundamental role in cell adhesion and communication, changes in HBP flux affect glycan structures and consequently influence epithelial morphology and EMT [155, 156]. GFPT-mediated O-GlcNAcylation and glycan synthesis are crucial for regulating the expression of both epithelial and mesenchymal markers [157–159]. Gene expression profiling reveals that GFPT2 is a metabolic signature of mesenchymal cancer cell lines, upregulated in EMT and prominent in metastatic tumors exhibiting EMT [160]. Furthermore, GFPT2 expression positively correlated with markers of cancer-associated fibroblasts (CAF) [137, 148], which play a role in cancer cell migration and invasion. In metastatic tumors, GFPT2 expression is also favorably associated with EMT-promoting transcription factors, including SNAIL, TWIST, and VIMENTIN [115, 131].

Transcriptomics profiling reveals upregulation of GFPT2 in mesenchymal stem cells within KRAS-driven lung cancer models [115]. GFPT has been linked to the aggressive and metastatic phenotype of cholangiocarcinoma [62], and in breast cancer, GFPT2 is highly expressed in mesenchymal cells and is notably associated with the claudin-low subtype [140]. Elevated GFPT2 levels are consistently observed from primary to metastatic leiomyosarcoma, underscoring its critical role in cancer metastasis [52]. GFPT2 is also implicated in stomatin-like protein 1 (STOML1)-driven liver metastasis of pancreatic cancer [161]. In gastric cancer, recent studies show that high GFPT2 expression correlates with increased tumor invasion and poor patient outcomes [148], while downregulation of GFPT1 in the same cancer type is similarly associated with EMT, invasion, and adverse prognosis [162]. Given the established links between GFPT isoforms and tumor progression, the contrasting roles of GFPT1 and GFPT2 in gastric cancer warrant further investigation.

### GFPT in cancer immune response and inflammatory signaling

The HBP serves as a critical communication axis between immune-inflammatory signaling and metabolism, with GFPT acting as a key player linking immune signaling and glucose metabolism, making it a potential target in immunotherapy. Recent studies reveal a direct relationship between GFPT expression and immune cell infiltration in tumors, where GFPT has been shown to promote T-cell exhaustion and increase inhibitory immune checkpoints such as PD-L1/PD-1 in the tumor microenvironment, suggesting an immunosuppressive role [131, 148, 163–165]. GFPT2, specifically, is associated with immunosuppressive cells like M2 macrophages and cancer-associated fibroblasts, contributing to immune evasion. For example, GFPT2-driven glutamine consumption disrupts mitochondrial function in macrophages and reduces their phagocytic activity. In EGFR-mutated NSCLC, targeted GFPT2 inhibition restores anti-tumor immunity by enhancing CD8 + T cell infiltration and reducing immune checkpoint activity [166]. Likewise, GFPT1 stabilizes PD-L1 via O-GlcNAcylation, which promotes IFNγ-induced PD-L1 activation and reduces T-cell and NK-cell efficacy against lung cancer cells [167]. Pharmacological GFPT1 inhibition, or blockade of glutamine utilization through DON treatment, heightens PDA sensitivity to anti-PD-1 therapy by diminishing hyaluronan levels and altering the extracellular matrix (ECM) [165]. These immunosuppressive functions of GFPT explain the correlation between high GFPT expression and poor patient outcomes.

In colorectal cancer, GFPT1 is connected to mitochondrial antiviral-signaling protein (MAVS) from early to late stages, supporting reprogrammed metabolic states seen in viral immune responses [168, 169]. Viral infections, such as respiratory syncytial virus (RSV), stimulate GFPT2 to drive HBP, leading to increased UDP-GlcNAc synthesis and viral glycoprotein production [47, 170]. Moreover, GFPT2 contributes to M2 macrophage polarization in PDA cells through the GFPT2-O-GlcNAcylation-YBX1 axis [171], and LPS-triggered inflammatory signaling induces GFPT2 expression via TLR4-FoxO1 in macrophages and TNF-NF-κB in NSCLC [51, 172]. These findings underscore the immunosuppressive and pro-inflammatory roles of GFPT in cancer, with therapeutic potential in modulating immune and inflammatory responses.

### Targeting GFPT in cancer

GFPT, the rate-limiting enzyme of the HBP and a central regulator of cellular UDP-GlcNAc levels, plays a pivotal role in cancer metabolism. Due to its essential function in glycosylation, GFPT has attracted increasing interest as a potential therapeutic target, particularly in cancer and metabolic diseases such as diabetes. A range of GFPT inhibitors—both synthetic and naturally derived—have been investigated, initially in the context of antimicrobial and antidiabetic therapies [173, 174]. These include analogs of fructose-6-phosphate or glucosamine-6-phosphate that target the isomerase domain, glutamine analogs that inhibit the glutaminase domain, and compounds that mimic the enzyme transition state [175–178]. Among these, glutamine analog N³-(4-methoxyfumaroyl)-2,3-diaminopropanoic acid (FMDP) demonstrates high selectivity for GFPT [179], whereas others such as DON (6-diazo-5-oxo-L-norluecin) and Azaserine (O-diazoacetyl-L-serine) exhibit broader activity against glutamine-dependent enzymes [180].

In cancer, therapeutic strategies targeting GFPT1 primarily involve glutamine analogs that inhibit glutamine-utilizing amidotransferases. Although multiple analogs have been evaluated for anticancer potential, only a subset—most notably DON and Azaserine—have been shown to directly inhibit GFPT activity [180, 181].

### Early GFPT1-targeting therapies in cancer

**Fig. 7:**
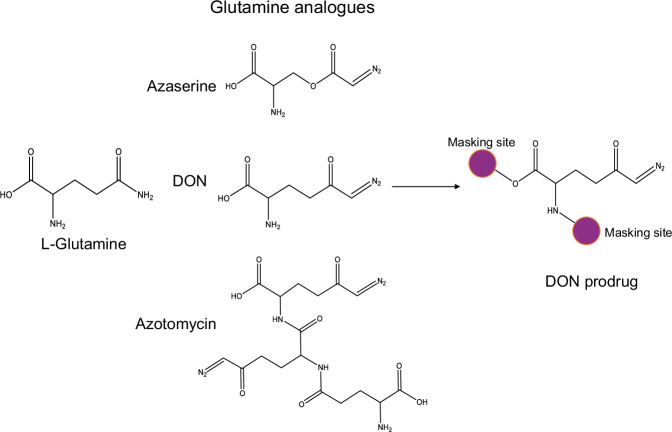
Inhibitors of GFPT. GFPT-targeting inhibitors investigated in cancer studies include the glutamine analogs Azaserine, DON, Azotomycin, and various DON-based prodrugs. In prodrug design, the α-carboxyl and amino groups of DON are masked with nonpolar, hydrophobic moieties to yield an initially inactive compound, which is subsequently bioactivated in vivo to release DON.

Among the earliest glutamine analogs explored for anticancer therapy, DON and Azaserine stand out as notable compounds that target glutamine-utilizing enzymes, including GFPT1. Both are naturally derived from *Streptomyces species* [182] and were identified over half a century ago during the intensive search for antibiotic compounds with antitumor properties [183, 184]. Structurally, DON and Azaserine are analogs of L-glutamine and differ only at the fourth atomic position: DON contains a carbon atom, whereas Azaserine features an oxygen atom (Fig. 7). DON is chemically synthesized through diazotization of the γ-carboxyl group of protected L-glutamic acid, following selective masking of its amino and carboxyl groups [185, 186]. In contrast, Azaserine is synthesized via O-diazoacetylation of L-serine [187, 188]. Early studies revealed that both compounds are highly polar, thermally unstable, and prone to degradation, necessitating stringent conditions to ensure reliable synthesis and yield [189, 190]. Moreover, the safety concerns associated with diazo compound synthesis have historically limited their commercial availability [191, 192]. However, recent breakthroughs in microbial genomics have identified the biosynthetic gene clusters responsible for DON and Azaserine production in *Streptomyces albus* and *Glycomyces harbinensis*, respectively [193, 194]. These findings open new avenues for sustainable, fermentation-based production of these diazo-containing glutamine analogs, offering a scalable and potentially safer alternative to chemical synthesis.

The diazo functional groups of DON and Azaserine confer high electrophilicity, a property central to their biological activity as irreversible inhibitors of glutamine-dependent amidotransferases [195]. These compounds covalently bind to the substrate-binding pockets of their target enzymes, resulting in permanent inactivation [196, 197]. Mechanistic studies suggest that, within the GFPT glutaminase domain, the inhibitor undergoes protonation—potentially facilitated by the thiol group of the catalytic Cys1 residue. This protonation event initiates a nucleophilic attack by the thiolate on the electrophilic diazonium group of the inhibitor, forming a covalent adduct and releasing molecular nitrogen (N_2_) [28]. The potent inhibitory properties of DON and Azaserine have demonstrated significant anticancer effects in multiple preclinical models. However, their acid lability and poor lipophilicity limit their pharmacokinetic properties—particularly their ability to cross the blood–brain barrier—thereby restricting their therapeutic efficacy against brain tumors such as glioblastoma [189].

Following promising preclinical results, DON and Azaserine entered clinical trials as early as the 1950s [198, 199]. Despite initial indications of therapeutic efficacy, clinical development was halted due to dose-limiting gastrointestinal toxicities, including nausea, vomiting, diarrhea, and mucositis, which were difficult to manage [184, 200]. Interest in DON resurfaced in the 1980s, spurred by the clinical success of Azotomycin—a naturally occurring compound composed of two DON molecules linked via an amide bond and a single glutamine moiety attached through its terminal amine group (Fig. 7) [184, 201]. Subsequent clinical studies showed that DON was generally well-tolerated in pediatric populations and exhibited promising outcomes when used in combination with pegylated glutaminase in adults with advanced, treatment-refractory solid tumor [202, 203]. However, clinical development was ultimately discontinued due to challenges in optimizing treatment regimens and dosing schedules, as DON-induced toxicity was found to be highly schedule-dependent and difficult to predict [199, 204].

### DON prodrugs

The recent recognition of cancer glutamine addiction, coupled with the notable antitumor potential of DON, has reignited efforts to repurpose DON with an improved therapeutic index. To address previous limitations—including poor plasma stability, low bioavailability, systemic toxicity, and inadequate lipophilicity for brain penetration—new DON prodrugs have been developed. These prodrugs are designed by masking both the carboxylate and amine functional groups of DON with various promoieties, such as alkyl esters, amino acids, or bulky hydrophobic groups, enabling in vivo bioactivation to release active DON [205]. The first generation of these optimized prodrugs, such as compound 5c, was engineered to enhance stability and facilitate delivery to the central nervous system (CNS) for potential treatment of brain tumors [205]. Building on this foundation, second-generation DON prodrugs—including JHU395, JHU083, and compound 6—incorporated tumor-targeting features and tumor-specific bioactivation, minimizing DON exposure in normal tissues, especially the gastrointestinal tract [198, 206–208]. This specificity was achieved by integrating promoieties cleavable by tumor-enriched enzymes, thereby restricting DON release to the tumor microenvironment. Given their robust preclinical efficacy (summarized in Table 3), several of these prodrugs have advanced toward clinical application. Notably, DRP-104, one of the most promising candidates, is currently undergoing clinical evaluation as both a monotherapy and in combination regimens for advanced solid tumors (NCT04471415) [209]. Furthermore, a recent study has introduced a hypoxia-activated DON prodrug, tailored to selectively target hypoxic tumor regions [210]. Intriguingly, combining this prodrug with a hypoxia-enhancing agent improved antitumor efficacy even in tumors with low baseline hypoxia, suggesting broader therapeutic potential irrespective of tumor microenvironmental oxygenation.

**Table 3:** Preclinical Features of DON Prodrug.

DON prodrug	Plasma stability	Distribution to tumor /CNS	Effect and activation	Tolerability and toxicity	References
Compound 5	Specie-specific stability	Specific for brain delivery. 10-fold higher than DON	Promising antitumor activity in brain tumors	Not Determined	[205]
JHU083	Stable	Tumor-specific and CNS delivery with micromolar brain concentration	Robust antitumor activity, immune activation, and apoptotic induction	Orally bioavailable and very tolerable. No significant gastrointestinal toxicity at the therapeutic dose	[208, 215]
JHU395	Stable	Tumor-specific and CNS delivery >2-fold tumor/plasma ratio compared to DON	Robust antitumor activity with apoptotic induction	Orally bioavailable and very tolerable. No significant gastrointestinal toxicity at the therapeutic dose	[207, 216]
Compound 6	Stable	Tumor-specific delivery. 6-fold higher tumor exposure of DON than plasma	Tumor-specific bioactivation with in vitro antiproliferative effect	Very Tolerable. Minimal DON release in G1 tissues and consequent limitation in G1 toxicity	[206]
Prodrug 1/2/3	Stable	Tumor-specific and CNS delivery	Tumor-specific bioactivation with antitumor effect	Tolerable. Minimal DON release in G1 tissues and limited toxicity at the effective therapeutic doses	[198]
DRP104	Stable in humans and in CES1^-/-^ mice	Preferential tumor delivery. 6-fold higher tumor exposure of DON than plasma	Tumor-specific bioactivation. Robust antitumor effect with complete tumor regression and immune activation.	Very tolerable. Negligible DON exposure to G1 and plasma. Reduced toxicity	[209]
Compound 11 (Optimized DRP104)	Stable in humans and CES1^-/-^ mice	Preferential tumor delivery. 3.6-fold higher tumor exposure of DON than plasma	Tumor-specific bioactivation with in vitro antiproliferative effect.	Not Determined	[217]
Azo-DON	Stable	Tumor-specific delivery	Hypoxia-specific bioactivation. Enhances antitumor immunity and apoptosis.	Dosage-dependent tolerability.	[210]

The chemicals that are detived from DON and considered as prodrug are listed here to demonstrate their properties and activities in cancer treatment.

Overall, compounds targeting GFPT1 exhibit promising therapeutic potential in cancer management. However, many of these agents also inhibit other glutamine-dependent enzymes, resulting in broad perturbations of cellular metabolism. For instance, while the glutamine analog FMDP selectively inhibits microbial GFPT orthologs, its clinical application has so far been restricted to antimicrobial settings. In addition, several small molecules that specifically inhibit the isomerase domain of GFPT or interfere with its interdomain interactions have been primarily explored in the context of diabetes and metabolic disorders [173]. Given their potent inhibitory activity, such compounds may hold untapped anticancer potential—particularly in malignancies characterized by high metabolic plasticity and upregulated hexosamine biosynthetic pathway (HBP) flux. Future efforts aimed at the strategic optimization and repurposing of these inhibitors for oncologic use could provide novel avenues for GFPT-centered translational research, offering new insight into metabolism-targeted cancer therapy.

### Differential roles of GFPT isoforms and concerns in translational study

Enzymes of the hexosamine biosynthetic pathway (HBP), yet they exhibit distinct biological roles that bear important implications for clinical and translational research. GFPT1 is ubiquitously expressed across multiple tissues, reflecting its role as the primary isoform mediating basal HBP activity. In contrast, GFPT2 displays tissue-restricted expression, suggesting a more context-specific regulatory function [34]. This divergence is also evident in their regulatory mechanisms. GFPT1, but not GFPT2, is subject to feedback inhibition by UDP-GlcNAc, a key mechanism for maintaining glycosylation homeostasis under steady-state conditions [37, 211]. Conversely, in cells where GFPT2 predominates, AMDHD2—a GlcNAc-6-P deacetylase—is required to regulate intracellular glycosylation flux [211]. The physiological relevance of GFPT1 is further underscored by its critical role in neuromuscular junction function, its association with congenital myasthenic syndromes, the presence of a muscle-specific isoform, and its essentiality for embryonic viability [35, 36, 212]. In cancer, the expression of GFPT1 and GFPT2 is differentially regulated under varying cellular stress conditions. For example, ER and nutrient stress induce GFPT1 through the activation of XBP1 and ATF4 [46, 78], whereas oxidative stress selectively induces GFPT2, potentially via receptor tyrosine kinase (RTK) signaling and NF-κB activation [77, 139, 140]. Although the precise rationale behind the conditional induction of these isoforms remains to be fully elucidated, the ER stress–mediated upregulation of GFPT1 highlights its primary role in maintaining glycosylation for protein folding and stability, whereas GFPT2 may serve stress-adaptive functions under distinct pathological contexts.

**Fig. 8:**
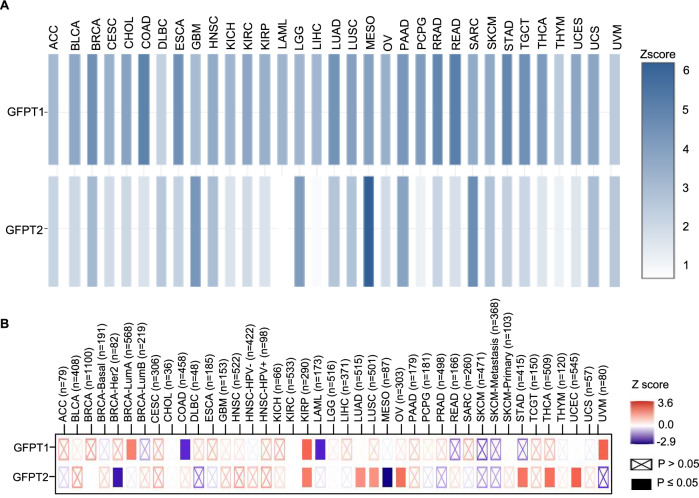
Comparative pan-cancer expression and tumor stage correlation of GFPT1 and GFPT2. **A** Comparative gene expression analysis of GFPT1 and GFPT2 across multiple TCGA tumor types was performed using the Gene Expression Profiling Interactive Analysis (GEPIA) tool [218]. Expression values are represented as Z-scores of the median Log_2_(TPM + 1) across tumor samples for each cancer type. **B** Correlation analysis between GFPT1 and GFPT2 expression levels and tumor stage across TCGA cancers was conducted using TIMER2.0 [219]. The heatmap displays the normalized regression coefficients derived from a Cox proportional hazards model for each gene (GFPT1 or GFPT2), indicating their association with tumor stage.

Comparative analyses of GFPT isoform expression across diverse tumor types reveal that GFPT1 is broadly and constitutively expressed, while GFPT2 exhibits more restricted, tumor-specific expression patterns (Fig. 8A). This supports the notion of GFPT1 as the basal metabolic isoform, with GFPT2 playing more specialized, context-dependent roles. The differential expression observed in cancer further suggests distinct biological functions for GFPT2 that are not redundant with those of GFPT1. Notably, accumulating evidence indicates that GFPT1 is more closely associated with tumor growth and proliferation, whereas GFPT2 correlates with tumor metastasis and progression [51, 97, 131, 161]. In support of this, expression analyses show that high GFPT2 levels are positively correlated with advanced tumor stages in multiple cancer types, a trend not observed for GFPT1 (Fig. 8B). Moreover, GFPT2 expression is enriched in EMT- and ECM-associated cellular populations, including mesenchymal cells, stromal cells, and cancer-associated fibroblasts (CAFs), further underscoring its role in invasion and metastatic dissemination [137, 148, 160, 213]. These isoform-specific functional differences pose a significant challenge for therapeutic strategies targeting GFPT in cancer. While GFPT1’s widespread expression raises concerns about potential off-target effects in normal tissues, GFPT2’s restricted expression profile and association with metastatic phenotypes positions it as a more attractive and potentially safer therapeutic target, particularly in aggressive and oxidative stress-rich tumors. However, the development of isoform-specific inhibitors remains difficult due to the high structural homology between GFPT1 and GFPT2 and the potential for compensatory function between the isoforms.

Additionally, the upstream regulatory role of GFPT in protein glycosylation poses a major challenge for its clinical translation as a therapeutic target. Inhibiting GFPT leads to a global suppression of protein glycosylation, a process essential for numerous cellular functions, thereby increasing the risk of off-target effects in normal tissues. Another key concern is the integration of GFPT within broader metabolic networks, including glycolysis and amino acid metabolism. This interconnectedness complicates the ability to isolate the specific contribution of GFPT1, as downstream effects may result from broader metabolic disruptions rather than GFPT inhibition alone. Furthermore, the lack of GFPT-specific inhibitors in preclinical studies raises concerns about misattribution of therapeutic effects, where observed outcomes may be erroneously interpreted as GFPT-specific due to concurrent off-target inhibition. In summary, GFPT plays a central role in cancer growth and progression. While several factors currently limit its feasibility as a direct therapeutic target, the isoform-specific functions of GFPT1 and GFPT2 present an opportunity to develop cancer subtype-specific biomarkers and may inform the design of more selective therapeutic strategies.

## Conclusion and perspective

The role of GFPT in cancer metabolism is increasingly recognized as a pivotal modulator linking nutrient stress response to tumor progression via the regulation of HBP. While traditionally GFPT has been associated with glycolysis and UDP-GlcNAc synthesis, evidence indicates that GFPT-mediated metabolic adaptation supports cancer cell survival, particularly under stress conditions such as nutrient deprivation and hypoxia. Although GFPT activity can be inhibited in glucose-starved cells, its expression is vital for cancer cell resilience, suggesting its potential as a therapeutic target for tumor types with high HBP dependency.

Despite the significant findings, much remains to be understood regarding the roles of GFPT in modulating cancer stemness, chemoresistance, and the interaction with other oncogenic pathways. Importantly, GFPT appears to facilitate EMT, invasion, and metastasis in part by enabling glycosylation of critical proteins. However, these mechanisms may vary by cancer type, highlighting the need for further research to elucidate GFPT’s role across different cellular contexts. Studies on the dysregulation and role of GFPT in cancer are mostly focused on GFPT-induced glycosylation; other non-conventional mechanisms like GFPT-induced glutamylation need to be explored.

Targeting GFPT and the HBP offers a promising strategy in cancer therapy, particularly as tumors with high GFPT expression are often more resistant to conventional chemotherapy. While glutamine analogs like DON have shown efficacy by inhibiting GFPT alongside other glutamine-dependent pathways, their broad action limits therapeutic specificity and increases toxicity. Future drug development should focus on optimizing GFPT-specific inhibitors to enhance the selectivity and reduce side effects in cancer treatments. Although prodrugs with better therapeutic index have been developed, their non-selectivity for GFPT still raises concern in translational GFPT research.
